# A Case of Undetected Neuroborreliosis in a 75-Year-Old Chinese Male

**DOI:** 10.1155/2018/6764894

**Published:** 2018-06-20

**Authors:** Jenny Lamichhane, Rohail Haider, Michael Bekkerman, Sean Tilford, Shireen Hijaz, Bikash Bhattarai, Rajanish Bobde

**Affiliations:** ^1^Department of Medicine, St. John's Riverside Hospital, 967 N. Broadway Yonkers, NY 10701, USA; ^2^Lake Erie College of Osteopathic Medicine, 1858 W. Grandview Blvd., Erie, PA 16509, USA; ^3^Interfaith Medical Center, 1545 Atlantic Avenue, Brooklyn, NY 11213, USA

## Abstract

Lyme disease is a multisystem infection caused by the spirochete *Borrelia burgdorferi* sensu stricto that manifests with characteristic symptoms in patients. Patients are identified based on their clinical symptoms and then diagnosed through enzyme-linked immunosorbent assay (ELISA), Western blot, and blood culture techniques. Here, we present the case of a 75-year-old, Northeast suburban resident complaining of unstable gait, high fevers, malaise, myalgia, and confusion. This patient's symptoms were nonspecific, and his lab titers and blood cultures were repeatedly negative during his stay. It was only late in the course of his treatment that blood titers and cerebrospinal fluid analysis were positive for Lyme IgG and IgM. He was treated with intravenous doxycycline and prescribed oral doxycycline on discharge, resulting in a full recovery. We express the need for physicians to consider Lyme disease in endemic patients presenting with nonspecific systemic signs.

## 1. Introduction

Lyme disease (LD) is a multisystem infection caused by the spirochete *Borrelia burgdorferi* sensu stricto in the United States, with other *B. burgdorferi* species as culprits in Europe and Asia [1]. The United States Centers for Disease Control and Prevention reported the incidence of Lyme disease to be 30,000 cases per year [2] but estimates that there are over 300,000 new cases of Lyme disease per year [2–4]. *B. burgdorferi* sensu stricto is the most common strain causing LD found in the Americas. It is most commonly transmitted via the *Ixodes scapularis* tick, and uncommonly via *Ixodes pacificus* [4, 5].

LD is divided into 3 stages: early localized, early disseminated, and late disseminated. Nonspecific symptoms, such as fatigue, anorexia, headache, neck stiffness, and myalgias, may appear at any stage [1]. Lyme neuroborreliosis (LNB) is a form of the disease present in either early or late disseminated stages and can occur days to months after the initial tick bite. The neurological presentation of LD occurs in 10–15% of cases [6] and can include radiculopathies, meningitis, facial nerve palsy, or other cranial nerve neuropathies [1, 2, 4, 7]. Between 10 and 20% of symptomatic patients may present with meningitis, meningoradiculitis, or meningoencephalitis [4, 5].

Diagnosis of LD begins with identifying clinical signs, then confirming with serological tests and imaging. The low number of spirochetes in clinical samples and antigenic variation make diagnosis very difficult [7]. Blood culture is also another method of testing for LD. Blood cultures for LD are positive in 50% cases with recognized erythema migrans [2]; however, this method has low sensitivity [6, 7], a long incubation of 8 to 12 weeks to confirm a negative result, and the use of special media and expertise. Blood culture is still the gold standard to confirm LD. PCR assays have similar sensitivities as blood culture but have more variation due to different methodology, gene targets, and primer sets used [7].

The medical literature is rife with cases of LD presenting without erythema migrans or other typical symptoms. Almoussa et al. described a case of LNB where the first manifestation of the disease was a cerebral ischemic stroke, nonspecific systemic signs (malaise, headache, and amnestic cognitive impairment), meningeal signs, and a tick bite 4 weeks prior. This patient's serology showed positivity for *B. burgdorferi* sensu stricto IgA and IgM, and appropriate treatment resulted in resolution of symptoms [1]. Shah et al. presented a case of third degree atrioventricular (AV) block and systematic signs in a patient without erythema migrans or other LD-typical signs. The AV block resolved after antibiotic treatment [8]. Burakgazi and Henderson described a case of optic neuritis with decreasing visual acuity and visual field defects, generalized rash, and other systemic signs as a first presentation of LD. Like the other cases, symptoms resolved when treated with LD specific antibiotics [9].

Here, we present a patient admitted to our hospital and diagnosed with neuroborreliosis who did not initially present with typical signs and symptoms of Lyme disease. The patient's labwork and diagnostic studies did not initially suggest LNB, but subsequent cerebrospinal fluid analysis after clinical decompensation confirmed the diagnosis.

## 2. Case Presentation

A 75-year-old Chinese male with a past medical history of hypertension, hyperlipidemia, coronary artery disease, diabetes mellitus, benign prostatic hyperplasia, and osteoarthritis arrived to our Emergency Department (ED) due to unstable gait. The patient stated that he was a resident of Yonkers, New York (NY). His only travel history involved a train ride to Flushing, NY, the day prior to presentation. The patient reported frequent walks in local parks around Yonkers, NY. At the time of initial examination, he denied headaches, dizziness, shortness of breath, back or chest pain, rashes, focal weakness, or loss of sensation. He had not noticed any ticks or tick bites on his skin in the past year. On physical exam, he was noted to have normal extraocular muscle movements, neurologic exam without focal deficits, and musculoskeletal, cardiac, and respiratory exams without abnormalities. His electrocardiogram showed normal sinus rhythm with a rate of 69 beats per minute. His labs revealed slightly decreased hemoglobin and hematocrit levels and thrombocytopenia along with increased bilirubin levels. His chest X-ray (CXR), rapid influenza swabs, blood cultures for bacteria, and urine analysis were negative. He was treated in the ED until he was hemodynamically stable and asymptomatic, after which he was then discharged.

He returned 4 days later complaining of worsening fevers since discharge, with a maximum recorded temperature of 105 degrees Fahrenheit (40.55°C), occasional rigors, chills, diaphoresis, diffuse myalgias, generalized weakness, malaise, confusion, and decreased appetite. The patient's gait was noted to be unstable with difficulty maintaining balance. On physical examination, the patient appeared lethargic. He was noted to be tachycardic at 98 beats per minute (bpm), to have a black discoloration of patient's tongue, and to have a recorded temperature of 102.8 degrees Fahrenheit (39.33°C). His blood pressure, respiratory rate, and the remainder of his neurological exam was normal. His CXR was significant for fullness in the right mediastinum, cardiomegaly, and unfolded aorta. His hemoglobin, hematocrit, and comprehensive metabolic panel remained stable from previous admission. It was noted that the patient had decreased white blood cell (WBC) count and severe thrombocytopenia on readmission (Table 1). Initial serum ELISA for Lyme disease, used as a screening tool, was negative. Magnetic resonance imaging (MRI) of the brain showed no plaque deposition, demyelination, or other acute pathologic processes. A computed tomography (CT) scan of the abdomen revealed a small aneurysmal dilatation of the thoracic aorta and splenomegaly with an acute appearing splenic infarct. He was started on ceftriaxone, ampicillin, and vancomycin to cover for a bacterial infection.

During the second night of hospitalization, the patient's heart rate spiked to over 200 bpm and he became hypotensive. He was transferred to the Intensive Care Unit (ICU) for management of severe hemodynamic instability and decompensated respiratory status. The patient's fever of unknown origin, encephalopathy, and negative initial screening tests prompted us to draw additional sets of serum tests for multiple possible bacterial, viral, and fungal etiologies. Unlike the initial serology, this new set yielded positive test results for the Lyme IgG and IgM ELISA test, Lyme IgM Western blot, and *Babesia microti* IgG and IgM antibody tests (Table 2). To confirm the diagnosis, a lumbar puncture (LP) was performed after patient's thrombocytopenia was corrected. During the LP procedure, the CSF was noted to be proteinaceous and congealed by the performing interventional radiologist. The LP resulted in positive tests for Lyme IgG and IgM antibody levels by Western blot, as well as a positive Western Blot for Lyme IgM (Table 3). Cytomegalovirus, Epstein-Barr virus, West Nile virus, and rheumatoid factor were not present. The patient was then started on doxycycline for the diagnosis of Lyme neuroborreliosis. Within 48 hours of doxycycline administration, the patient clinically improved, his mentation returned to baseline, and his gait returned to normal. The patient was treated for 5 days and was discharged on an oral course of doxycycline for a total of 21 days.

Here we have presented a patient who had no characteristic clinical signs of LD, whose main symptoms were a high fever, encephalopathy, diffuse myalgia, and tachycardia. Blood culture, urine culture, and CSF gram stain were negative. An MRI of the brain and CT of the chest/abdomen showed no source of infection. The single diagnostic modality yielding concrete results was the patient's CSF Lyme serology.

## 3. Discussion

Lyme disease can be divided into the early localized, early disseminated, and late disseminated stages. Early localized disease occurs 3–30 days after tick exposure and is characterized by erythema migrans (EM) at the site of tick bite [1, 2, 4, 7]. Between 60 and 80% of patients develop EM [4–6]. The early disseminated stage usually occurs days to months after the tick bite and may include secondary EM lesions [2], acute carditis, nervous system signs, or oligoarticular arthritis [1, 4, 7]. Lyme neuroborreliosis can initially present in the early disseminated stage [1, 2, 4, 7]. The late disseminated stage occurs months to years after exposure. It may include joint and/or nervous system complications. In our case, due to our patient's vague presentation, it was difficult to determine the chronological stage of the disease, although it can reasonably be deduced that he was between the early and late disseminated stages.

Infection with *B. burgdorferi* sensu stricto should be considered in patients presenting with nonspecific systemic signs, myalgias, and very high fevers, especially if patients reside in endemic areas. In our case, we presented a patient whose initial screening test was negative for Lyme disease. A lumbar puncture was necessary to confirm Lyme disease.

Lyme neuroborreliosis usually presents with cranial nerve palsies, lymphocytic pleocytosis, and radiculopathy [2, 6, 10]; the most common presentation is facial nerve palsy [2, 4]. Meningitis, sensory and motor radiculoneuropathies like mononeuritis multiplex, and meningoencephalitis [2, 4, 5] may present in LNB; strokes, intracranial hemorrhages, and sinus thrombosis are rare but possible complications in LNB [1]. Our patient did not have clinical or pathological evidence of overt meningitis. However, his unsteady gait could be a result of associated distal motor neuropathy. In order to diagnose LNB, IgM and/or IgG antibodies to *B. burgdorferi* sensu stricto are tested in the CSF of patients, as suggested by Shah et al.; however, those reports also identified serum IgM/IgG positivity [10]. Our case clearly provides evidence for the possibility that CSF analysis for Lyme neuroborreliosis may be the only positive confirmatory test. Physicians should be aware of the characteristic and noncharacteristic signs of LNB in order to quickly perform an LP and establish the diagnosis such that antibiotic therapy can promptly begin.

## Figures and Tables

**Table 1:** Laboratory results of the patient obtained on initial presentation and subsequently on readmission 4 days later, revealing a significantly reduced WBC count and a worsening thrombocytopenia.

Hematology	Initial presentation to Emergency Department	Return to Emergency Department 4 days later	Normal ranges
White blood cells (K/mm^3^)	4.3	3.8	4.0–10.0
Red blood cells (K/mm^3^)	4.09	4.14	4.00–5.60
Hemoglobin (g/dL)	11.3	11.3	11.7–16.9
Red cell distribution width (%)	14.3	14.3	11.9–15.9
Platelet count (K/mm^3^)	77	36	134–434
Neutrophils (%)	48	45	42.8–82.8
Lymphocyte (%)	27	33	8–40
Monocyte (%)	17	10	3.8–10.2
Band neutrophils (%)	7	9	0–10

**Table 2:** Serologic results of the patient obtained on initial evaluation during his second admission and again after the patient began to clinically decompensate in the intensive care unit.

Serology	Initial results on readmission to hospital	Repeat results after ICU admission	Normal ranges	Methods applied
*Babesia microti* IgG	—	1 : 20 (high)	<1 : 10	Immunofluorescence assay
*Babesia microti* IgM	—	1 : 160 (high)	<1 : 10	Immunofluorescence assay
Lyme screen IgG and IgM	0.89	3.25 (high)	0.00–0.90	ELISA
Lyme disease IgG/IgMs	0.82	3.17 (high)	0.00–0.90	ELISA
Lyme IgM Western blot	—	Positive	Negative	Western blot
West Nile virus IgG	—	Negative	Negative	ELISA
West Nile virus IgM	—	Negative	Negative	ELISA
*H. influenza* antigen type B	—	Negative	Negative	Latex agglutination
*N. meningitidis* antigen	—	Negative	Negative	Latex agglutination
*S. pneumoniae* antigen	—	Negative	Negative	Immunochromatography assay
GrpB *Streptococcus* antigen	—	Negative	Negative	Latex agglutination
Rheumatoid factor	—	<10.0 (negative)	0–15	Latex immunoturbidimetry
Antinuclear antibody	—	Negative	Negative	Immunofluorescence assay

The only screening test initially performed was Lyme ELISA, which returned negative.

**Table 3:** Cerebrospinal fluid analysis of our patient in the intensive care unit was positive for elevated total protein as well as the majority of IgG and IgM antibody fragments on Western blot.

CSF analysis	Results	Normal ranges
Glucose (mg/dL)	74	50–80
Total protein (mg/dL)	158 (high)	15–45
Albumin (mg/dL)	0.08	—
Venereal disease research lab	<1 : 1	<1 : 1
Lyme IgG Western blot	Positive	Negative
Lyme IgG Ab 18 kDa	Positive	Negative
Lyme IgG Ab 23 kDa	Positive	Negative
Lyme IgG Ab 28 kDa	Positive	Negative
Lyme IgG Ab 30 kDa	Positive	Negative
Lyme IgG Ab 39 kDa	Positive	Negative
Lyme IgG Ab 41 kDa	Positive	Negative
Lyme IgG Ab 45 kDa	Positive	Negative
Lyme IgG Ab 58 kDa	Positive	Negative
Lyme IgG Ab 66 kDa	Positive	Negative
Lyme IgG Ab 93 kDa	Negative	Negative
Lyme IgM Western blot	Positive	Negative
Lyme IgM Ab 23 kDa	Positive	Negative
Lyme IgM Ab 39 kDa	Positive	Negative
Lyme IgM Ab 41 kDa	Positive	Negative
Cysticercosis Ab	0.04	<0.34
CMV IgG Ab	<0.20	Negative
HSV I and II DNA PCR	Negative	Negative
Toxoplasma IgG Ab	Negative	Negative
Gram stain	No growth	No growth
